# Semantic Wavelet-Induced Frequency-Tagging (SWIFT) Periodically Activates Category Selective Areas While Steadily Activating Early Visual Areas

**DOI:** 10.1371/journal.pone.0144858

**Published:** 2015-12-21

**Authors:** Roger Koenig-Robert, Rufin VanRullen, Naotsugu Tsuchiya

**Affiliations:** 1 School of Psychological Sciences, Faculty of Biomedical and Psychological Sciences, Monash University, Melbourne, Australia; 2 CNRS, UMR5549, Centre de Recherche Cerveau et Cognition, Faculté de Médecine de Purpan, 31052 Toulouse, France; 3 Université de Toulouse, Centre de Recherche Cerveau et Cognition, Université Paul Sabatier, 31052 Toulouse, France; 4 Decoding and Controlling Brain Information, Japan Science and Technology Agency, Chiyoda-ku, Tokyo, Japan, 102–8266; Kyoto University, JAPAN

## Abstract

Primate visual systems process natural images in a hierarchical manner: at the early stage, neurons are tuned to local image features, while neurons in high-level areas are tuned to abstract object categories. Standard models of visual processing assume that the transition of tuning from image features to object categories emerges gradually along the visual hierarchy. Direct tests of such models remain difficult due to confounding alteration in low-level image properties when contrasting distinct object categories. When such contrast is performed in a classic functional localizer method, the desired activation in high-level visual areas is typically accompanied with activation in early visual areas. Here we used a novel image-modulation method called SWIFT (semantic wavelet-induced frequency-tagging), a variant of frequency-tagging techniques. Natural images modulated by SWIFT reveal object semantics periodically while keeping low-level properties constant. Using functional magnetic resonance imaging (fMRI), we indeed found that faces and scenes modulated with SWIFT periodically activated the prototypical category-selective areas while they elicited sustained and constant responses in early visual areas. SWIFT and the localizer were selective and specific to a similar extent in activating category-selective areas. Only SWIFT progressively activated the visual pathway from low- to high-level areas, consistent with predictions from standard hierarchical models. We confirmed these results with criterion-free methods, generalizing the validity of our approach and show that it is possible to dissociate neural activation in early and category-selective areas. Our results provide direct evidence for the hierarchical nature of the representation of visual objects along the visual stream and open up future applications of frequency-tagging methods in fMRI.

## Introduction

Neural processing in the ventral visual pathway is fundamental for object recognition in primates. Standard models of visual processing have proposed that different hierarchical processing steps are needed to extract information from simple features in early visual areas to invariant category representations in higher-level areas within the visual pathway [1,2]. While neurons in early visual areas (i.e., V1/V2) principally respond to the physical properties of the visual input [3–6], neurons in the inferotemporal cortex show invariant responses to object categories [7–9].

To distil the invariant neural responses to object categories, we previously designed a novel method called SWIFT (semantic wavelet-induced frequency-tagging), which allows manipulating the high-level image semantics without changing the principal low-level image features [10]. Specifically, SWIFT periodically scrambles a natural image in the wavelet domain, modulating semantic content of the image at a fixed temporal frequency while conserving the image’s local luminance modulation, local spatial frequency and global contrast. As a result, neurons in the early visual areas, which are tuned to these image’s physical features, are expected to be steadily activated, while neurons in the high-level visual areas, tuned to object categories, are expected to respond periodically at the tagging-frequency. Previous results combining SWIFT with electroencephalography (EEG) have shown that SWIFT is sensitive to conscious high-level visual representations of visual objects [10], however, due to poor spatial resolution of EEG, it has been unclear whether or not SWIFT selectively and specifically activates the high-level category-selective areas without concomitant periodic activation in other areas.

Here, we measured blood-oxygen-level dependent (BOLD) signals with fMRI to extend the previous study [10] by assessing the spatial pattern of activation elicited by SWIFT at high spatial resolution. To match the slow dynamics of BOLD, we used low-frequency SWIFT to tag the responses elicited by faces, scenes and objects. We tested two key predictions regarding the spatial profiles of neuronal activation. First, we predicted that SWIFT would activate the high-level category-selective areas as selectively and specifically as standard functional localizers [11–13]. Second, we predicted that SWIFT would constantly activate the low-level visual areas while periodically activating the high-level visual areas. As a benchmark, we compared the results of SWIFT with those obtained by the classic, block-design functional localizer.

We validated the assumptions of the SWIFT method and provided a proof of concept, confirming these predictions for faces and scenes using both a fixed statistical threshold as well as criterion-free analyses. Furthermore, we found that the higher in the hierarchy, the more voxels SWIFT activated in a frequency-tagged fashion: steady and flat responses in V1 and V2, strong and periodic responses in category-selective areas, and intermediate responses in V3 and V4. The localizer, on the other hand, activated low- and higher-level areas more similarly compared to SWIFT. SWIFT category-related activations are consistent with models that propose gradually refined representations of visual objects along the visual hierarchy.

## Materials and Methods

### Participants

Experimental procedures were approved by the Monash University Human Research Ethics Committee (CF12/2542–2012001375). Written consent was taken from nine paid participants (3 female, mean age: 29.2, standard deviation = 7.6), one of them was excluded due to failure in maintaining adequate levels of arousal during the experiment. Seven out of the remaining 8 participants were tested in Session 1 and all 8 participants were tested in Session 2. Sessions 1 and 2 were conducted on different days, several weeks apart. Session 1 and 2 contained different versions of the SWIFT experiments (see below). In addition to the SWIFT experiments, Session 2 contained the functional localizer as well as a retinotopic mapping experiment. All participants had normal or corrected to normal vision.

### Functional and structural MRI parameters

Scanning was performed at the Monash Biomedical Imaging facility, Melbourne, Australia, in a 3 Tesla MRI scanner (Siemens Magnetom Skyra) using a 32-channel Head Coil. T2*-weighted functional images were acquired using a gradient-echo echoplanar imaging (EPI) sequence (TR = 2.46s, TE = 30ms, flip angle = 90°, matrix size = 64x64, voxel size = 3x3x3mm, acceleration factor = 2). Forty-four contiguous sagittal slices were acquired covering the whole brain. For the structural MRI, 256 T1-weighted sagital slices covering the whole brain were acquired using magnetization prepared rapid acquisition gradient echo (MP RAGE) sequence (TR = 1.9s, TE = 2.43ms, flip angle = 9°, matrix size = 256x256, voxel size = 0.6x0.6x0.6mm). Two structural MRIs were averaged to obtain the final structural image. For one participant who was tested only in Session 2, two structural MRIs acquired in Session 2 were averaged. For the rest of participants, one structural MRI obtained in Session 1 and another in Session 2 were averaged. Additional magnetic field (B0) mapping was acquired for off-line distortion correction of EPI sequences due to B0 inhomogeneity.

### SWIFT stimuli

**Fig 1:**
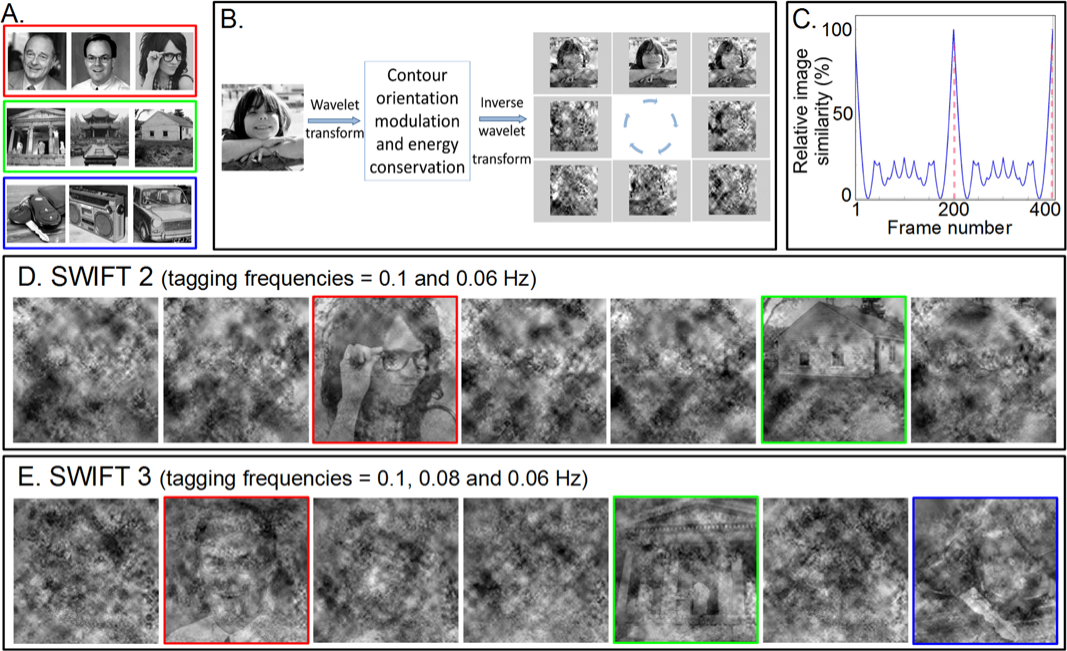
The SWIFT paradigm. (A) Images used in the experiments: three faces (red rectangle), scenes (green) and man-made objects (blue). (B) The SWIFT principle. Cyclical local-contour scrambling in the wavelet-domain allows smoothly modulating the image's semantic content at a fixed temporal frequency (the tagging-frequency) while conserving its principal physical attributes and avoiding strong onset effects (see Materials and Methods). This allows dissociating neural mechanisms related to low-level feature extraction (engaged equally across the stimulus presentation) from mechanisms related to object recognition (periodically engaged at the tagging-frequency). (C) Relative image similarity between the unscrambled image and each frame of the SWIFT movie over two cycles (see Materials and Methods). The image becomes recognizable around the frames where the unscrambled content is revealed (marked with red dashed lines) while, for most of the remaining frames, the image is highly degraded. (D) and (E) Exemplar frames used for SWIFT. (D) In SWIFT 2, two SWIFT movies were superimposed. Face and scene images were modulated at two different tagging-frequencies (0.06 or 0.1Hz). Unscrambled frames are highlighted with colored frames (not shown in the actual experiment). Importantly, within a given pair, all frames in the SWIFT movie segment are matched in the principal local physical properties, since face's and scene's principal low-level properties are present at any given frame. (E) In SWIFT 3, three SWIFT movies were modulated at 0.06, 0.08 and 0.1Hz. Here, face's, scene's and object's principal low-level properties are present at any given frame.

We used 3 natural grayscale images per each category of human faces, scenes and man-made objects, all downloaded from the Internet (Fig 1A). We used Google Images (https://www.google.com/imghp) to find images with “free to use, share or modify, even commercially” usage rights. Semantic wavelet-induced frequency-tagging (SWIFT) was used to modulate pictures' semantic information at a fixed temporal frequency while conserving the principal physical features of the image [10]. In short, SWIFT periodic movies were created by cyclic wavelet scrambling in the wavelet multi-scale domain (Fig 1B). In the wavelet domain, strength of horizontal, vertical and diagonal orientations of the original image at each location and scale was represented by a 3D vector. In addition to the original vector, two new vectors with random orientations but the same length were defined for each location and scale. The unique isoenergetic circular path described by the 3 vectors was used to modulate local contour orientation cyclically. As a result, the original image was rendered into a sequence of its wavelet-scrambled versions, which conserved the principal physical features of the original image (local luminance modulation, global contrast and local spatial frequency). We created the SWIFT movies for the experiments reported here in MATLAB (The MathWorks Inc., Natick, MA) using a function (available in [10]). We used the following parameters: independent scrambling cycles = 3, number of harmonics = 8, frames per cycle = 200, wavelet decomposition levels = 9.

One cycle of a SWIFT movie goes from the original image, through its fully scrambled version and to the original image again. The transitions are smooth, minimizing transient effects (see S1 Movie). Fig 1C shows the time course of the degree of image scrambling as the relative image similarity to the original picture over two cycles of 200 frames each. At frame number x, the relative image similarity (RIS) was defined as:

$${RIS}_{x}=\sum_{c=1}^{n}(1-\frac{{|W}_{c}o-W_{c}x|}{n})\cdot \;100$$

Where W_*c*_o and W_*c*_x are the wavelet coefficients at the scale/position *c* at frame number *o* and *x* for the original and the rendered image, respectively, for *n* number of wavelet coefficients. Thus, for a given wavelet coefficient, when the original image corresponds to the rendered image, the relative difference between them is zero (|W_*c*_o − W_*c*_x| = 0), and the RIS = 100. When the relative difference between the original and the rendered image is maximal (|W_*c*_o − W_*c*_x| = 1), the RIS thus becomes 0.Importantly, the original image is only briefly recognizable at each cycle while, for the rest of the frames, the image is unintelligible, as is clearly seen in the examples in Fig 1D and 1E, as well as in S1 Movie. (As such, we cannot define the exact duration for presenting the original image. However, see our psychophysics experiments to address this issue). As shown in Fig 1C, the wavelet scrambling does not follow a sinusoidal function but it is more similar to a u-shaped function, which is a result of the introduction of several harmonically related modulation frequencies in the wavelet domain. Small peaks in RIS (Fig 1C) are the results of partial phase alignment across some of the harmonically modulated wavelet components. RIS reaches 1 only when phases of all the harmonically related wavelet coefficients align at frame 200 and 400 (in Fig 1C), which we call *semantic-onset* (Fig 1D and 1E, colored rectangles).

### SWIFT experiments

We performed two SWIFT experiments. In the first experiment, which we call “SWIFT 2”, we presented 2 categories of images (i.e., a face and a scene) simultaneously in the same stream using alpha blending (50%) with one category tagged at 0.1Hz and the other at 0.06Hz (Fig 1D). In the second experiment, which we call “SWIFT 3”, we presented 3 categories (i.e., a face, a scene and an object), simultaneously in the same stimulus using alpha blending (33.3% for each category), tagged at 0.1, 0.08, and 0.06Hz (Fig 1E). These tagging-frequencies were chosen to fit the slow temporal dynamics of the BOLD response [14,15]. Simulation results using the canonical BOLD response revealed that this frequency range (i.e., <0.1Hz) was optimal in terms of signal-to-noise ratio for the TR used in the experiment (data not shown). Note that by overlying multiple SWIFT movies for different image categories in the same stimulus we preserved the local spatial frequency content, global contrast and local luminance modulation across frames. In other words, for a given pair of images in SWIFT 2, the principal physical features of faces and scenes were present in all frames, while for a given trio of images in SWIFT 3, the principal physical features of faces, scenes and objects were present in all frames.

In both SWIFT experiments, a 9min run was divided into three contiguous movies of 3min duration, each of which was composed of a particular pair or trio of images for SWIFT 2 and SWIFT 3. Three such segments were concatenated such that each new segment started from the frame number following the last frame of the previous segment, thus preserving the phase of the SWIFT cycles (relative to the semantic onset) throughout the block. For example, in a block with faces tagged at 0.1Hz, the original face pictures were always shown every 10 seconds, irrespective of a change in the face exemplar. Note that while there are differences in the low-level physical properties across the three segments, these differences will be reflected at a very low frequency (0.0056Hz), which is not harmonically related to the tagging-frequencies tested here and unlikely to affect our results. The tagging frequencies for each category were randomized and balanced across runs. The phase of the scrambling cycle for each category-stream was selected (randomly from frame number 30 to 100 out of possible 200) to prevent the appearance of the original images at the onset of the run. Six runs were shown for each participant, three for each SWIFT 2 and 3.

During SWIFT 2, participants were asked to fixate at the center of the display and attentively look at the movie. During SWIFT 3, in addition to the fixation instruction, participants performed a detection task; they pressed a button on a response box when a dim gray dot appeared on top of the SWIFT movie. The dot appeared at random times and locations (from 13 to 20 times during the 9min run).

### Functional localizer experiment

The same nine images used in the SWIFT experiments (Fig 1A) were used in the functional localizer experiment. In addition, their box-scrambled versions (256 parts, one scrambled version per image) were presented in a block design [16–22]. A block of a given category (faces, scenes, objects or their scrambled counterparts) was presented for 20s followed by 15s of fixation. Each image was presented on the screen for 0.75s and replaced by a gray screen with a fixation cross for 0.25s. Participants performed a one-back task both with natural and scrambled images, pressing a button on the response box when they detected the repeat of the image. Only 40% of blocks contained image repeats, while the number of the repeats varied randomly from 1 to 3 per block. The order of the categories was randomized across blocks. Each run lasted 7m15s; it began with 15s of fixation, followed by 12 blocks of image-presentation and fixation (35s). One run of 12 blocks were composed of 2 repeats of 6 conditions (3 categories x intact vs. scrambled). A total of 3 runs were performed for each participant.

### Phase-encoded retinotopic mapping

To determine the boundaries of visual areas from V1 to V4 for each participant, we used the phase-encoding method [23,24]. Double wedges containing dynamic colored patterns cycled through 10 rotations in 10min (retinotopic stimulation frequency = 0.033 Hz). To ensure deployment of attention to the stimulus during the mapping, participants performed the same detection task as used in SWIFT 3 (i.e., pressing a button upon seeing a gray dot anywhere on the wedges). The program for this experiment was downloaded from Samuel Schwarzkopf's tutorial (http://sampendu.wordpress.com/retinotopy-tutorial/). One run was performed for each participant.

### Scanning experiment procedures

Each session started with a structural MRI sequence. In Session 1, 3 runs of SWIFT 2 (9min) were performed as well as other experiments that are not reported here. The entire session lasted about 1.5h. In Session 2, after one run of retinotopic mapping (10min), SWIFT 3 (9min) and functional localizer (7min15s) were alternated, each performed 3 times. The entire session lasted about 1h. In all functional experiments a fixation cross was presented at the center of the display and participants were required to fixate on it throughout. In between the runs, rest periods were allocated as required by the participants. Stimuli were back projected through the magnet bore using an RGB video projector located outside the scanning room.

### Preprocessing

Functional MRI data from all experiments were preprocessed using SPM8 (Wellcome Department of Cognitive Neurology, London, UK, www.fil.ion.ucl.ac.uk/spm/). Preprocessing consisted in slice-timing correction, motion artifact correction, unwrapping (including B0 inhomogeneity correction), spatial smoothing using 3mm full width at half maximum (FWHM) Gaussian kernel and high-pass temporal filtering (filter width 128s).

### BOLD signal time course within region of interests (ROIs)

**Fig 2:**
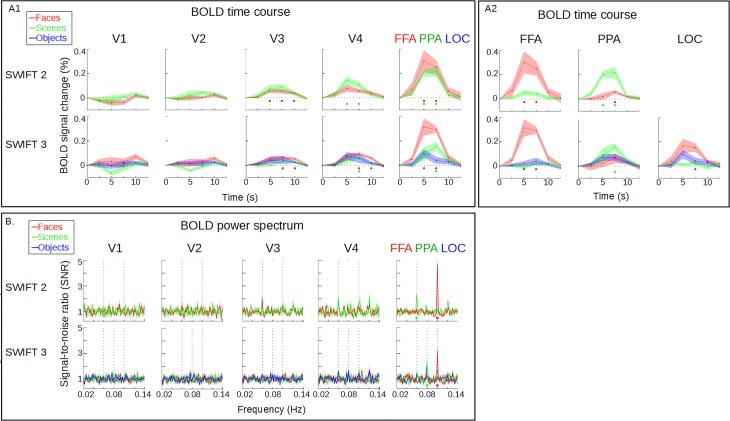
BOLD signals time courses and power spectra. (A.1) In each panel, BOLD time courses are shown, with time 0 corresponding to the onset of the unscrambled frame for SWIFT (up to 12.5 s). The BOLD signals were averaged within each retinotopic area (V1, V2, V3, and V4) and functionally-defined ROI (FFA, PPA and LOC). Responses to faces, scenes and objects are represented by red, green and blue lines, respectively. SWIFT elicited increasingly stronger frequency-tagged BOLD responses along the visual hierarchy, showing steady and flat time courses in the early visual areas (V1/V2), while significant deviations from baseline (colored squares) are present from V3 onwards. Maximal SWIFT responses were found in the functionally-defined ROIs, with significant responses for faces and scenes in SWIFT 2 and 3 but not for objects. Shaded area represents SEM across participants. (A.2) Same as A.1 but showing responses only in functionally-defined ROIs (FFA, PPA and LOC) for both SWIFT 2 and 3 experiments. Faces and scenes frequency-tagged with SWIFT elicited significant responses in the FFA and the PPA respectively. Objects responses in the LOC did not reach statistical significance, while faces responses did, likely due to the overlap between the occipital face area (OFA) and the LOC. (B) BOLD power spectra for SWIFT 2 and 3. We normalized the spectra by power at the neighboring frequencies to obtain the signal-to-noise ratio (SNR, see Materials and Methods). Dashed lines represent the three tagging-frequencies (0.06, 0.08 and 0.1Hz). For visualization purposes, we present the spectra in a subset of experiments (i.e., SWIFT 2 with faces and scenes tagged at 0.1 and 0.06Hz and SWIFT 3 with faces, scenes and objects tagged at 0.1, 0.08 and 0.06Hz). Significant responses (colored squares) at the tagging-frequencies were present in the FFA and the PPA for faces and scenes, respectively, in both SWIFT 2 and 3. Shaded area represents SEM across participants.

We converted the raw BOLD signal time course of each voxel into % signal change by normalizing it to the global mean for the voxel. Then we aligned at time 0 the semantic onsets. Finally we averaged the aligned normalized time course across voxels within each ROI (see below for the definitions of ROIs) and across SWIFT cycles (Fig 2A).

### SWIFT frequency-tagged responses

We chose to analyze SWIFT responses in the frequency domain over other alternatives (e.g., event related GLM analysis) for two main reasons. First, it allows validating the SWIFT frequency-tagging approach in fMRI, which will be useful for future SWIFT studies (see Discussion) and, second, it will allow comparing more directly the current results with other SWIFT results analyzed in the frequency domain using diverse neuroimaging modalities (e.g., EEG, iEEG).

In the frequency-tagging EEG literature [25–27], noise is defined as power at the non-tagging frequencies and signal-to-noise ratio is defined as relative increase of power at the tagging-frequencies. This definition removes any need of the resting condition that is typically required in the standard fMRI protocol.

Time series (9min, 216 volumes) from the SWIFT experiments were transformed into the frequency domain using a Fast-Fourier Transform (FFT) algorithm (fft.m) implemented in MATLAB. For every voxel, the power at the tagging-frequencies (signal) was then compared to the power at the neighboring frequencies (noise). The noise consisted of 22 frequencies, sparing the tagging-frequencies (0.0464, 0.0483, 0.0501, 0.0520, 0.0538, 0.0650, 0.0668, 0.0687, 0.0705, 0.0724, 0.0742, 0.0854, 0.0872, 0.0891, 0.0910, 0.0928, 0.0947, 0.1058, 0.1077, 0.1095, 0.1114 and 0.1132 Hz). A p-value for each voxel was obtained by comparing the power at the tagging-frequency (0.1, 0.08 or 0.06 Hz) with the power at the noise frequencies using a two-tailed two-sample t-test. Signal-to-noise ratio (SNR) for each voxel was defined as the ratio of the power at a given frequency divided by the average power in a 0.0266 Hz band (14 bins) surrounding it (Fig 2B).

### Functional localizer responses

**Fig 3:**
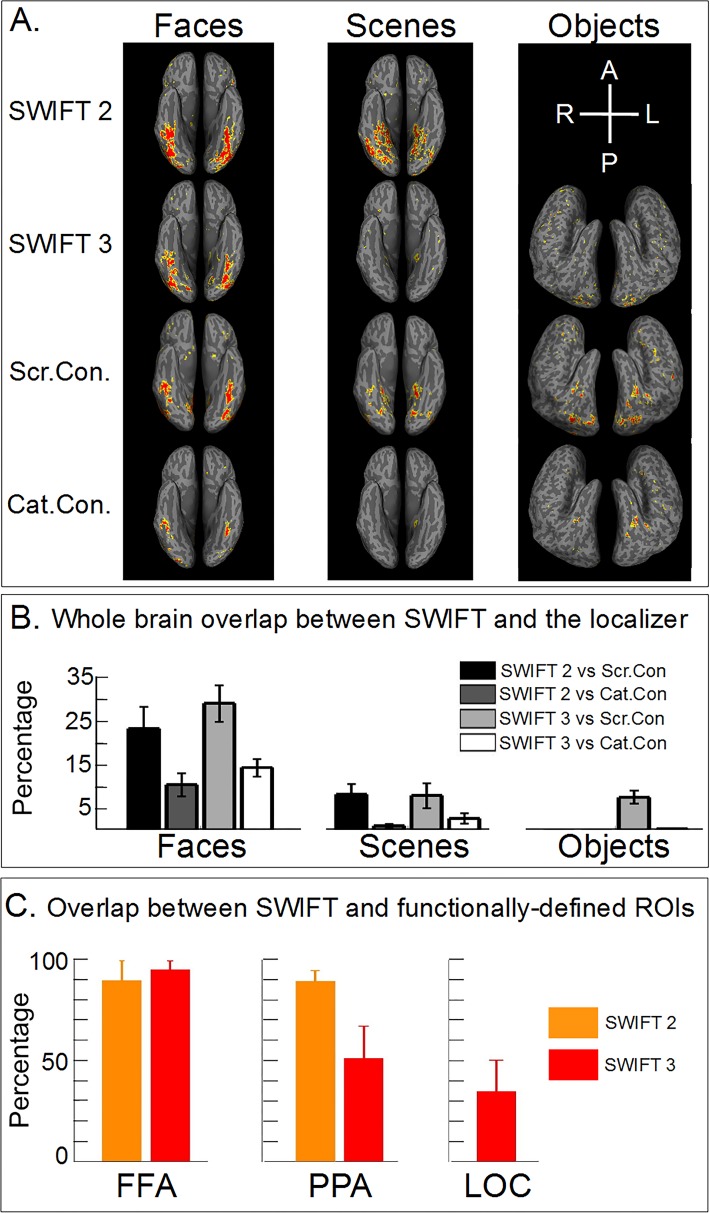
Spatial profile of activations for SWIFT and the localizer. In all panels of this figure, we used p<0.001 uncorrected as the statistical threshold. (A) Surface representation of category-selective activations for a representative participant. Ventral and lateral cortical surfaces are shown for faces/scenes and objects, respectively. (B) Overlap as the percentage of the voxels that were activated by SWIFT, which was also activated by the localizer across the entire cortex (see Materials and Methods for details). (C) Overlap as the percentage of the voxels in functionally-defined ROIs that was also activated by SWIFT. Orange and red bars represent SWIFT 2 and 3 respectively (see Materials and Methods for details). Error bars represent SEM across participants. Abbreviations: A for anterior, P for posterior, R for right, L for left, Scr. Con. for scramble-contrast, Cat. Con. for category-contrast.

We analyzed the localizer data using the general linear model (GLM) in SPM8, contrasting blocks of natural images from one category with those from another category in "category-contrast" analyses or with the corresponding scrambled images in "scramble-contrast" analyses (e.g., Fig 3). For category-contrast analyses, we used “faces > objects” [17,28] “scenes > objects” [29,30], and “objects > scenes” [31] for faces, scenes and objects, respectively.

### Retinotopic ROIs

Phase-encoded retinotopic mapping data was analyzed using the Fast-Fourier Transform (FFT) in MATLAB. The FFT was applied voxel-wise across time points. The complex output of the FFT contained both the amplitude and phase information of sinusoidal components of the BOLD signal. The phase information at the frequency of stimulation (0.033Hz) was extracted, using its amplitude as threshold (≥2 SNR, calculated as in the analysis of SWIFT data explained above) and overlaid them on each participant's cortical surface reconstruction obtained using Freesurfer [32,33]. We manually delineated boundaries between retinotopic areas on the flattened surface around the occipital pole by identifying the voxels showing phase reversals in the polar angle map, which represents the horizontal and vertical visual meridians. In all participants, we clearly defined five distinct visual areas: V1, V2, V3d, V3v and V4; throughout this paper, we merge V3d and V3v and label them as V3.

### Definitions of ROIs for category-selective areas

To define ROIs for category-selective areas, we used 3 distinct strategies: based on either functional activation in high-level visual areas, anatomy alone, or conjunction by SWIFT and the functional localizer. We used these definitions to reduce potential confounds and biases in the interpretation of the results.

#### Functionally-defined ROIs

Were defined based on the category contrast GLM analyses (see above) of the localizer data, with manual selection of voxels that showed activations at threshold p = 0.001, uncorrected, bilaterally in the fusiform, parahippocampal and lateral occipital cortex for faces, scenes and objects, respectively. For each participant, the fusiform face area (FFA), the parahippocampal place area (PPA) and the lateral occipital complex (LOC) were clearly defined as face-, scene- and object-selective ROIs.

#### Anatomically-defined ROIs

Were defined based on anatomical location of the voxels. Anatomical labels were defined by Freesurfer's automatic parcellation on a participant-by-participant basis [34,35]. We defined the fusiform ("G_oc-temp_lat-fusifor" in Freesurfer), parahippocampal ("G_oc-temp_med-Parahip") and middle occipital cortex ("S_oc_middle_and_Lunatus" and "G_occipital_middle") as the minimal anatomical areas that typically contain face-, scene- and object-selective voxels, respectively.

#### Conjunction-defined ROIs

Were defined as the anatomical regions in which the mean BOLD signals over all the voxels within the ROI showed significant activation in both the localizer and SWIFT. As candidate ROIs, we initially considered 4 retinotopic regions, defined by the retinotopy experiment for each subject, and 74 automatically labeled anatomical regions. To test the significance of activation in the localizer, we compared the mean BOLD time courses of a given ROI in a category-contrast manner as in the GLM analysis (e.g., faces vs scenes for faces). If the time course contained any time points that showed positive (e.g., faces > scenes for faces) and significant responses (two-sample t-test, corrected by FDR at q = 0.05), we proceeded to the next step. Next, to test the significance of activation in SWIFT, we tested if the mean time course during either SWIFT 2 or 3 contained any time points that showed significant differences from the average BOLD signal over time (one-sample t-tests, corrected by FDR at q = 0.05). If a given ROI passes both of the tests, we defined it as a part of conjunction-defined ROI. We thus obtained the conjunction ROIs for faces as 'S_occipital_ant', 'S_oc_middle_and_Lunatus', 'G_oc-temp_lat-fusifor' and 'G_and_S_occipital_inf'; for scenes as 'V4', 'G_oc-temp_lat-fusifor', 'S_oc_sup_and_transversal', 'S_oc_temp-med_and_Lingual' and 'G_oc-temp_med-Parahip'; and for objects as 'V4', 'G_oc-temp_lat-fusifor' and 'S_oc-temp_med_and_Lingual'.

#### Dorsal and ventral anatomical clusters

To quantify the degree of activation elicited by SWIFT and the localizer along the visual pathway, we defined two anatomical clusters including dorsal and ventral visual areas. For the dorsal cluster (Dsl), we grouped four automatically labeled ROIs parcellated using Fresurfer including lateral occipital areas ('S_oc_sup_and_transversal', 'G_occipital_middle', 'S_oc_middle_and_Lunatus' and 'S_occipital_ant'). For the ventral cluster (Vtl), we grouped five automatically labeled ROIs ('G_and_S_occipital_inf', 'S_oc-temp_lat', 'G_oc-temp_lat-fusifor', 'S_oc-temp_med_and_Lingual' and 'S_collat_transv_post') including inferior-temporal areas.

### Overlap analysis

We used two overlap analyses to assess the degree of similarity between the spatial patterns of activation by SWIFT and the localizer. We first used a whole brain analysis (Fig 3B) in which we quantified the percentage of SWIFT-activated voxels that were also activated by the localizer as follows:

$${Ov}_{whole\;brain}=\left(\frac{\left|{SW}_{all}\cap {FL}_{all}\right|}{\left|{SW}_{all}\right|}\right)\;\cdot \;100$$

Where *SW*_*all*_ and *FL*_*all*_ are the sets of voxels activated across the whole brain by SWIFT and the functional localizer, respectively and | * | denotes the number of elements in a set *.

In a second analysis, we focused on the similarity of the spatial patterns of activation within the prototypical category-selective areas (Fig 3C). We thus quantified the percentage of the functionally-defined ROIs (i.e., FFA, PPA and LOC) that were activated by SWIFT as follows:

$${Ov}_{category\;areas}=\left(\frac{\left|FD\cap {SW}_{all}\right|}{\left|FD\right|}\right)\cdot \;100$$

Where *FD* is the set of voxels in a given functionally-defined ROI.

### Receiver operating characteristic analysis

The two key questions we address in this paper are 1) to determine the selectivity and specificity of SWIFT activation within the category-selective ROIs and 2) to test if SWIFT preferentially engages the high-level visual areas while keeping constant activation in the early visual areas. To answer these questions, we need a quantification scheme that allows us to characterize relative sensitivities between two cortical areas. Furthermore, we need to compare such quantity obtained from SWIFT with the localizer, which depend on different statistical procedures (e.g., t-tests in power spectra and the GLM analyses). How can we compare the results of these in a fair manner?

To overcome the challenges, we developed a method based on a concept of receiver operating characteristics (ROC) analysis [36]. In ROC analysis, the proportion of ‘hits’ or positive responses in signal trials is compared with the proportion of ‘false alarms’ or positive responses in no-signal trials. For a given criterion, a combination of the proportion of hits and false alarms defines a point’s x,y coordinate. By varying the criterion from highly stringent to very liberal, points from [0,0] to [1,1] are connected to form an ROC curve. The area under the ROC curve (AUC) quantifies the discriminability of signal presence from absence in a criterion-free manner.

**Fig 4:**
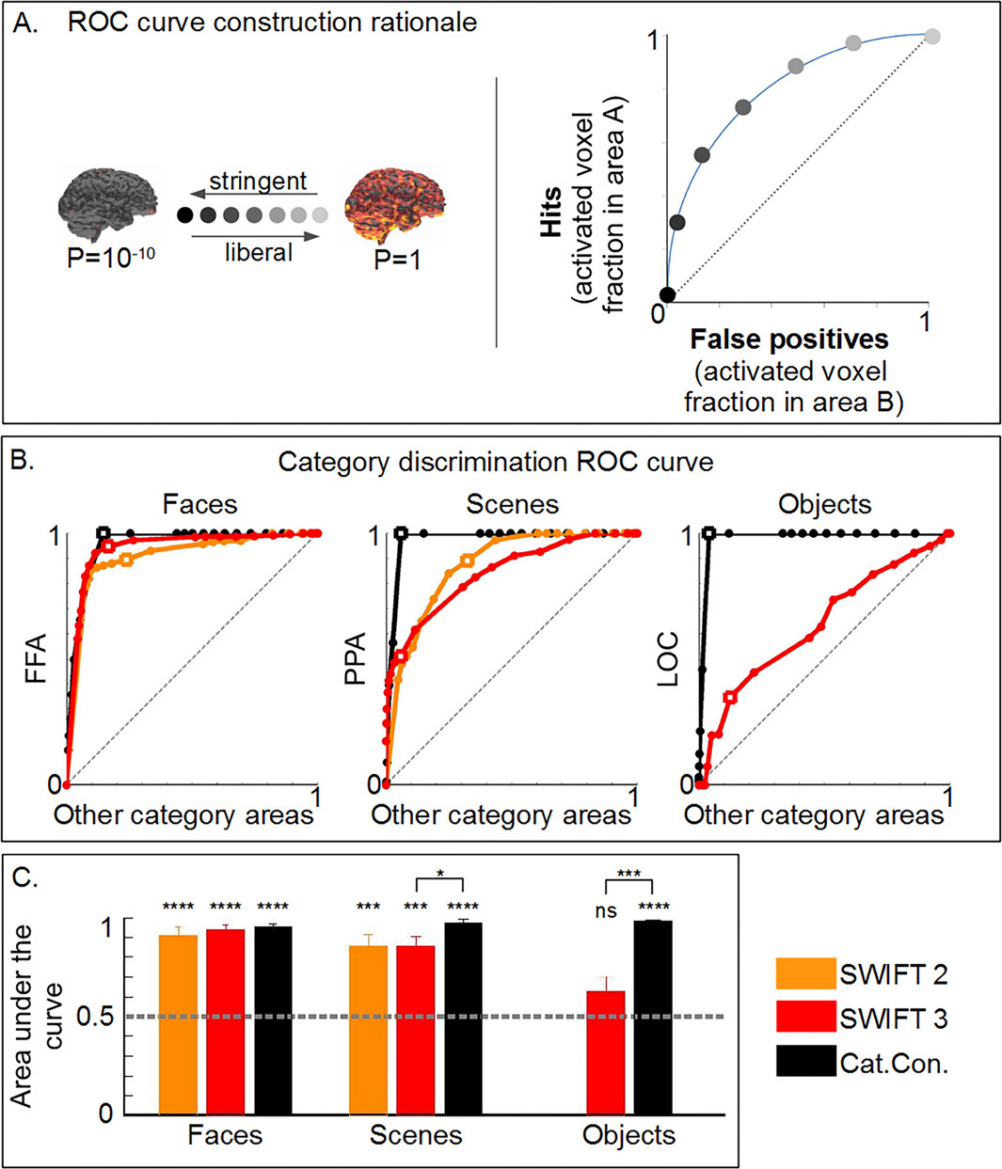
Criterion-free analyses of the selectivity and specificity of functionally-defined ROIs for SWIFT and the localizer. (A) The ROC analysis. By varying the p-value threshold from stringent to liberal, curves are constructed by connecting the dots that indicate the proportion of the voxel that pass a given threshold. (B) The ROC analyses with hits and false alarms defined as the proportion of significant voxels in the functionally-defined ROIs that are selective for each category (e.g., FFA for faces) and in the remaining ROIs which are not selective for a given category (e.g., PPA and LOC for faces. See main text for details). Orange, red, and black lines indicate the ROC curves constructed using the data in SWIFT 2, 3 and the localizer, respectively. Squares on the curves represent p = 0.001. The squares always locate with hits = 1 for the localizer because we defined ROIs as such. (C) Area under the curves (AUC). Asterisks above the bars indicate either significant differences from AUC = 0.5 (one-sample t-test) or differences among different conditions (two-sample t-test). n.s., *, **, *** and **** represent p>0.05, <0.05, <0.01, <0.001 and <0.0001.

For our purpose, we varied a p-value threshold from very stringent (p<10^−10^) to very liberal (p = 1) in 20 discrete steps (ten steps in a log scale from p<10^−10^ to p<10^−1^, and another ten steps in a log scale from p<10^−0.9^ to p = 1), while we computed the proportion of the voxels that exhibited p-values lower than the threshold (Fig 4A). For example, we can construct an ROC curve, which represents a relative sensitivity for V1 and face ROI in a particular experiment, by plotting the proportion of the voxels in V1 and face ROI that are below a certain p-value threshold in the x- and y- axes, respectively. The AUC reaches 1 if all voxels in face ROI, and no voxels in V1, are below a particular p-value threshold. The AUC is 0 if all V1 voxels and no FFA voxels are below a particular p-value threshold. The AUC is around 0.5, when the same proportions of the voxels are below over a range of p-value thresholds.

Thus, our ROC method provides a way to compare relative sensitivity and specificity of a pair of ROIs in two experiments in a criterion-free manner, avoiding arbitrary selection of a particular statistical threshold.

To characterize this novel approach, we have run a simulation shown on S1 Supporting Information. The simulation shows that our approach is well suited to capture relative category selectivity among ROIs.

### Psychophysics experiment

Because SWIFT reveals the unscrambled image gradually over frames (Fig 1C), it is not possible to define its exact onset and duration. To estimate the latency and vividness of perception of each category in the SWIFT experiments we developed a psychophysics experiment. We examined 32 participants who have never participated in any experiment that involved SWIFT. In each trial (9 in total), one of the SWIFT movies was shown, which started from a random frame number from 30 to 100 (out of possible 200). For example, for the face category, each of the three face pictures was presented as a target embedded in either SWIFT 2 (paired with a scrambled scene) or SWIFT 3 (paired with a scrambled scene and a scrambled object). To avoid ambiguity, the SWIFT movies for the non-target categories were looped among highly scrambled frames (from frame 50 to 150). Thus, SWIFT movies for the non-target categories never showed up as the original image. Half of the participants were randomly assigned to see the face as embedded in SWIFT 2, and the other half in SWIFT 3. The same random assignment was performed for each of three scenes. All object images were presented as SWIFT 3.

Participants were asked to press a button as fast as they confidently saw any image from one of the three categories. Reaction time (RT) was defined as the difference in time between the button press and the moment when the fully unscrambled frame was (going to be) presented. RT was negative if participants identified the image category before the frame reaches the fully unscrambled frame. Each trial finished at the moment the button was pressed or 18s after the start of the trial. After each trial, participants reported which category of the image they saw (i.e., a three alternative choice) and rated its vividness in a scale of 1 to 4. Participants were instructed to give vividness rating based on the relative number of details of the image that they perceived.

## Results

### SWIFT reveals categorical information periodically, while conserving low-level image properties

We employed semantic wavelet-induced frequency-tagging (SWIFT) to modulate the semantic content of natural images of human faces, scenes and man-made objects (Fig 1A). SWIFT local-contour orientation scrambling preserves a natural image's local spatial frequency content, local luminance modulation and global contrast across frames, while it periodically reveals the semantic content (Fig 1B, for details see [10]). Constructed as such, standard models of visual processing would predict that SWIFT activates neurons in the low-level visual areas at a constant level throughout while it activates neurons in the high-level areas periodically, in a frequency-tagged manner. In each SWIFT scrambling cycle, the original image is briefly presented (Fig 1C, dotted red line) while, for most of the remaining frames, the image is unintelligible. By alpha blending, we superimposed two or three SWIFT movies that are constructed with images from different categories (Fig 1D and 1E for SWIFT 2 and SWIFT 3, respectively) tagged with different frequencies.

### Perceptual latency and visibility of natural images scrambled by SWIFT

Behavioral analyses of a psychophysics experiment confirmed that participants were able to reliably perceive faces, scenes and objects. Across categories, participants tended to respond at a comparable speed, yet they perceived faces more vividly than scenes and objects. Specifically, in terms of RT, there were no main effects of categories nor the number of movies superimposed (both p>0.05, unbalanced two-way ANOVA); mean RT±SEM for faces were -0.37±0.05s and -0.2±0.1s for SWIFT 2 and 3; for scenes -0.79±0.34s and -1.1±0.4s for SWIFT 2 and 3; and for objects -0.65±0.17s. In terms of vividness, unbalanced two-way ANOVA revealed a significant main effect of categories (p<0.0001) but not of the number of movies superimposed (p>0.05), with faces perceived more vividly; mean vividness±SEM for faces were 2.8±0.1 and 2.8±0.1 for SWIFT 2 and 3; for scenes 2.0±0.2 and 1.5±0.5 for SWIFT 2 and 3; and for objects 1.4±0.2. No significant differences were found between SWIFT 2 and 3 within a given category. Note that these results depend on the exact choice of the stimuli set. Some aspects of the fMRI results reported in this article might reflect the perceptual characteristics of the particular stimuli set used (see Discussion).

### SWIFT tags by frequency high-level areas, but not low-level visual areas

Fig 2A shows the normalized time courses of BOLD signals in retinotopic areas as well as the functionally-defined ROIs. For both SWIFT 2 and 3, significant differences from the average BOLD response (one-sample two-tailed t-test, corrected at FDR q = 0.05) were found in V3 and FFA for faces as well as V4 and PPA for scenes, while no significant activations were found for objects. As predicted by standard models of visual processing, SWIFT activations increased from early visual areas to category selective areas, suggesting progressive category-selectivity along the visual hierarchy. Importantly, no significant frequency-tagged activations were found in V1 and V2.

Fig 2B shows the same BOLD signals in the SWIFT experiments now represented in the frequency domain. For visualization purposes, here we present the average spectra of a subset of trials across subjects, that is, the SWIFT 2 trials where faces and scenes are tagged at 0.1Hz and 0.06Hz, respectively (Fig 2B, top row), and the SWIFT 3 trials where faces, scenes and objects were tagged at 0.1, 0.08 and 0.06Hz, respectively (Fig 2B, bottom row). Comparable results were obtained using other trials with other combinations of tagging-frequencies for each category (not shown).

The frequency-domain analysis showed that the signal at the tagging-frequencies (dotted lines) are significantly greater than noise (one-sample one-tailed t-test, corrected at FDR q = 0.05), in the functionally-defined ROIs (in FFA and PPA for faces and scenes, red and green squares, but not in LOC for objects), while no signals were identified at the tagging frequencies in any of the retinotopic areas from V1 to V4. This result thus confirms that SWIFT frequency-tags BOLD signals in high-level category-selective areas, while keeping constant activation in early visual areas.

### Spatial profile of activation by SWIFT and the localizer: whole brain analyses

The first main question we addressed was whether the spatial extent of activation of SWIFT was as selective and specific, at least, as the classic functional localizer. Fig 3A shows significantly activated voxels at the p<0.001 threshold (uncorrected) by SWIFT and the localizer overlaid onto a representative participant's cortical surface. Other subjects showed qualitatively comparable results (not shown). Note that significance was assessed by t-test in the frequency-domain for SWIFT and by the GLM contrast in the time domain for the localizer (see Materials and Methods for details). Notice that our choice of an uncorrected statistical threshold (i.e., p<0.001) is mainly for illustration purposes. Our main conclusion does not rely on this particular uncorrected threshold, as we will show in detail with our criterion free ROC analyses (see subsequent sections).

Face activations by SWIFT were predominantly found along the fusiform gyrus (Fig 3A) as well as on the anterior part of the lateral occipital cortex (not visible in the ventral view shown on Fig 3A) which were concordant with the face selective cortical regions known as the fusiform face area (FFA) and the occipital face area (OFA) reported in the literature [17,37]. Face activations by the localizer were also mainly located in the fusiform and lateral occipital cortex, with the category-contrast functional localizer showing more restrained activations than the scramble-contrast functional localizer.

Scene activations by SWIFT 2 were found in the parahippocampal, the fusiform and the lingual cortex, while, for the SWIFT 3 experiment, the activations were more restricted to the parahippocampal gyrus (Fig 3A, see Discussion). Activations in the parahippocampal gyrus were concordant with the scene selective cortical region known as parahippocampal place area or PPA [29,31]. The spatial pattern of activation for SWIFT 2 was similar to that for the scramble-contrast localizer, revealing the parahippocampal and the fusiform gyri. The pattern for SWIFT 3 was similar to that of the the category-contrast localizer, confined mostly in the parahippocampal gyrus.

Objects activations by SWIFT 3 were scattered across different areas, with no consistent patterns across participants; we observed activations from the posterior lateral occipital cortex to the anterior middle temporal gyrus (Fig 3A) and also in the ventral areas such as the fusiform gyrus (not visible in the lateral view shown on Fig 3A).

### Spatial overlap of activation between SWIFT and the functional localizer across the whole brain

Next, we quantified the degree of spatial overlap between SWIFT and the localizer. We defined the overlap as the percentage of significant voxels across the whole brain according to the category- or scramble- contrast localizer that was also activated by SWIFT (see Materials and Methods for details). The overlap was significantly higher (p<0.05, two-sample two-tailed t-test) between SWIFT and the scramble-contrast localizer than between SWIFT and the category-contrast. This was the case for all categories and SWIFT 2 and 3, except for scenes when presented with two other categories (p>0.05). This was expected because SWIFT can be considered as a form of the scramble contrast (see Discussion). The overlap was generally low (0–27%), indicating that SWIFT and the localizer activated largely non-overlapping sets of the voxels across the whole brain.

### Spatial overlap of activation between SWIFT and the functional localizer in functionally-defined ROIs

Next, we confined the above spatial overlap analyses within the functionally-defined ROIs (FFA, PPA and LOC) to examine whether SWIFT can activate the same set of voxels that are localized in a standard neuroimaging procedure.

Fig 3C shows the percentage of significant voxels in the high-level visual areas according to the category-contrast localizer (i.e., functionally-defined ROIs) that was also activated by SWIFT, both at threshold p<0.001 (see Materials and Methods for details). Across the categories, SWIFT was able to activate a high percentage of the voxels within the functionally-defined ROIs; the percentages were remarkably high for the FFA (mean±SEM were 89.4±9.7% and 94.7±4.3% for SWIFT 2 and 3) and for the PPA in SWIFT 2 (89.0±5.3%) but were moderate for the PPA in SWIFT 3 (51.0±15.8%) and for the LOC (34.7±15.3%). This confirms that SWIFT periodically activates most of the voxels in the category-selective areas for faces and scenes as defined by the classic localizer.

### Criterion-free assessment of the spatial overlap of activation in the high-level visual areas

The results in Fig 3 depended on the particular statistical threshold which is usually used in neuroimaging studies (i.e., p<0.001, [38]). Depending on the threshold the proportion of the voxels that were activated by both SWIFT and the localizer varied. To test the generality of our claim, we developed a criterion-free analysis, employing a concept of receiver operating characteristic (ROC) analysis from the signal detection theory (see Materials and Methods for details). The ROC analysis addressed a concern arising from the overlap analysis in Fig 3B and 3C. Although SWIFT was able to activate the voxels that were activated by the localizer, it did not rule out a possibility that an extent of the voxels that SWIFT activated might have been more extensive and unspecific than the localizer. The specificity of the extent of significant activation was captured by the ROC analysis.

Fig 4A explains the concept of ROC analysis. For the ROC curve, we defined 'hits' as the proportion of voxels activated in the functionally-defined ROI for a given category (FFA, PPA or LOC, for faces, scenes and objects) and 'false positives' as the proportion of voxels activated in the other ROIs selective to other categories (i.e., PPA and FFA for faces and scenes respectively in SWIFT 2, and PPA+LOC, FFA+LOC and FFA+PPA for faces, scenes and objects in SWIFT 3 and the localizer). Fig 4B shows the resulting ROC curves, for faces, scenes and objects, comparing SWIFT 2, SWIFT 3 and the category-contrast functional localizer (orange, red and black curves, respectively). Each point on the curve represents a different statistical threshold (from p<10^−10^ to p = 1), with white squares representing the point with p<0.001 for both hits and false positives. AUC value of 0.5 means two ROIs were activated equally across a range of thresholds.

Because the FFA, PPA and LOC were defined at p<0.001 using the category-contrast localizer, all the ROC curves reach 1 in y-axis (i.e., 'hits' = 1) at p<0.001 (Fig 4B, white squares). The localizer’s AUC±SEM (Fig 4C) for faces, scenes, and objects, were 0.95±0.02, 0.97±0.02, and 0.98±0.00; one-sample, two-tailed t-test against 0.5 resulted all in p<0.0001 (Fig 4C), showing that the localizer’s activations were category specific within category-selective areas, as expected from previous studies [17,29–31].

We quantified the selectivity and specificity of SWIFT within the functionally-defined ROIs using the AUC (red and orange curves and bars in Fig 4B and 4C). SWIFT showed very high AUC values for faces (AUC of 0.91±0.04 and 0.94±0.03, for SWIFT 2 and 3, both p<0.0001). This means that SWIFT faces periodically activated nearly identical sets of voxels as the localizer within FFA without activating much voxels within PPA or PPA+LOC, regardless of the statistical threshold chosen; AUCs were not different between SWIFT and the localizer (two-sample, two-tailed t-test: both p>0.05). For scenes, the AUC values were also very high (0.86±0.06 and 0.86±0.05 for SWIFT 2 and 3, both p<0.001), yet AUC for SWIFT 3 was significantly lower than that for the localizer (p<0.05). Finally, for objects, SWIFT 3 AUC was 0.63±0.07, not statistically different from 0.5 (p>0.05) and was much lower than the AUC for the localizer (p<0.001).

All in all, both the fixed threshold (p<0.001, in Fig 3) as well as the criterion-free ROC analyses (Fig 4) confirmed that SWIFT, especially for faces and scenes, periodically activates high-level category-selective areas as selectively and specifically as the classic functional localizer.

### SWIFT reveals an increase in neuronal tuning for categories along the visual hierarchy while the functional localizer does not

Next, we addressed the second main question of this paper; whether SWIFT would constantly activate the low-level visual areas while periodically activating the high-level visual areas.

Using the same significance threshold at p<0.001, we calculated the proportion of voxels that are activated by SWIFT or the localizer along the visual pathways in an ascending order of the visual hierarchy (i.e., V1, V2, V3, V4, dorsal (Dsl) and ventral areas (Vtl)). To discard contributions to the variance that are due to individual differences and differences between the methods (i.e., SWIFT and the localizer), we normalized the proportion of voxels activated in each area by dividing it by the average proportion of voxels activated in V1 and V2. This allowed us to estimate the level of category-specific activation along the visual pathways, relative to that in V1 and V2, which are expected to show minimal category-specific responses.

**Fig 5:**
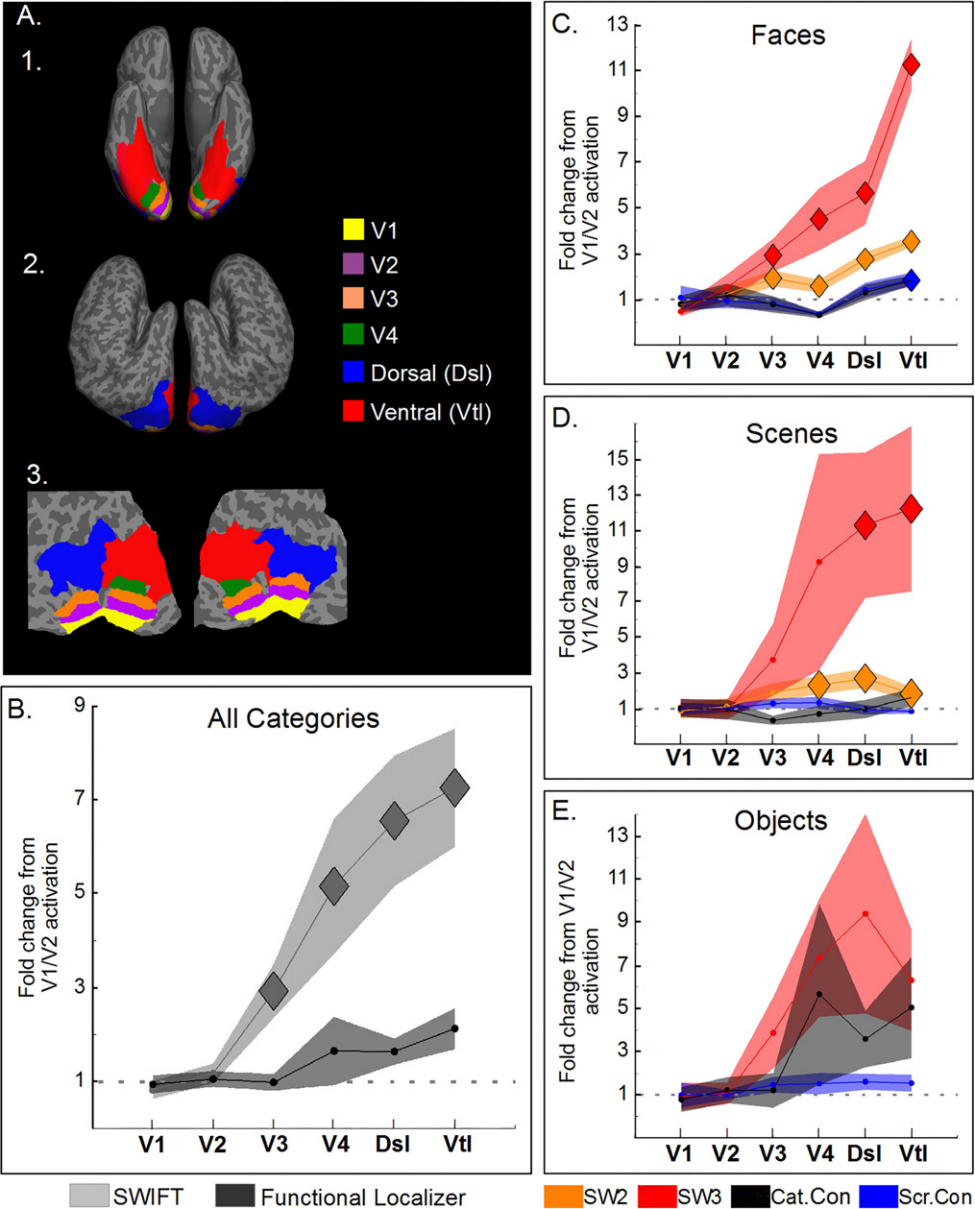
SWIFT increasingly activates areas along the visual pathway. (A) Cortical surface of a representative participant showing ROIs used in the analysis (see Materials and Methods). A1 Ventral view. A2. Lateral view. A3. Flattened occipital pole view. (B-E) Normalized proportion of activated voxels (as the fold change respective to V1/V2 activations, threshold at p<0.001) across different cortical areas hierarchically sorted: V1, V2, V3, V4, dorsal (Dsl) and ventral areas (Vtl). (B) Normalized proportion of activated voxels for all categories and experiments averaged, separately for SWIFT and the localizer. (C, D and E) for faces, scenes and objects, respectively. The proportion increases along the pathway in SWIFT 2 and 3 (orange and red) while it is uniform for the localizer with category- and scramble-contrast (black and blue). Shaded areas correspond to SEM across participants. Diamonds represent proportions significantly different from 1 (corrected at FDR q = 0.05).

On average, the normalized proportion of the activated voxels increased along the hierarchy for SWIFT, but not for the localizer (Fig 5B). When averaged across all categories for SWIFT 2 and 3, SWIFT clearly activated more and more voxels along the visual hierarchy; the normalized proportion was significantly above 1 for V3 onwards (diamonds, one-sample one-tailed t-test, corrected at FDR q = 0.05). This was clearly not the case for the localizer (averaged for the category and the scrambled contrasts for all categories), resulting in a flat line, where none of the points were significantly different from 1.

On a closer look at SWIFT 2 and 3 separately for each category (Fig 5C–5E), the normalized proportion increased along the hierarchy; they were significantly different from 1 for faces (from V3 onwards for SWIFT 2 and 3) and scenes (from V4 onwards for SWIFT 2 and from the dorsal areas onwards for the SWIFT 3), while, for objects, the normalized proportion was not different from 1 (p>0.05). The localizer (blue and black lines in Fig 5C–5E), in contrast, did not activate voxels in the high-level areas more than in V1/V2, except for the scramble-contrast activations in ventral areas for the face category.

### Criterion-free assessment of the relative activation between low- and high-level visual areas between SWIFT and the localizer

Finally, we re-assessed the above conclusions about relative activation of the low- and high-level visual areas obtained at the p<0.001 threshold using the criterion-free ROC analyses. This procedure is important due to the distinct experimental designs and statistical procedures for SWIFT and the localizer.

To quantify the relative activation between low- and high-level areas as AUC of the ROC curves, we defined 'hits' and 'false positives' as the proportion of significant voxels in high-level category-selective areas and V1/V2, respectively.

In the first analysis, we defined the high-level category-selective areas as the functionally-defined ROIs obtained with the localizer as we did for the previous ROC analysis (Fig 4). (We do not report the results in this paragraph as a figure). The results indicated that SWIFT preferentially activated the ROIs rather than V1/V2 showing high AUC values for faces (mean±SEM 0.93±0.04 and 0.97±0.01, both p<0.0001, one-sample two-tailed t-test against 0.5), for scenes (0.89±0.05 and 0.93±0.02, p<0.001 and p<0.0001) and for objects (0.76±0.06, p<0.01). These AUC values were comparable to those for the localizer: the localizer preferentially activated the ROIs than V1/V2 showing high AUC values for faces, scenes and objects (0.97±0.01, 0.97±0.02 and 0.99±0.00, all p<0.0001). The AUCs for faces and scenes were statistically indistinguishable between the localizer and SWIFT (p>0.5), while the AUC for objects was significantly greater in the localizer than in SWIFT 3 (p<0.01).

Although this first analysis supported the claim that SWIFT discriminates between low- and high-level visual areas, we needed to compare SWIFT and the classic functional localizer in a manner that the category-selective areas definition was not dependent only on the functional localizer results. We thus defined the high-level category-selective areas in two ways that are neutral in the selection of the voxels for both SWIFT and the localizer.

**Fig 6:**
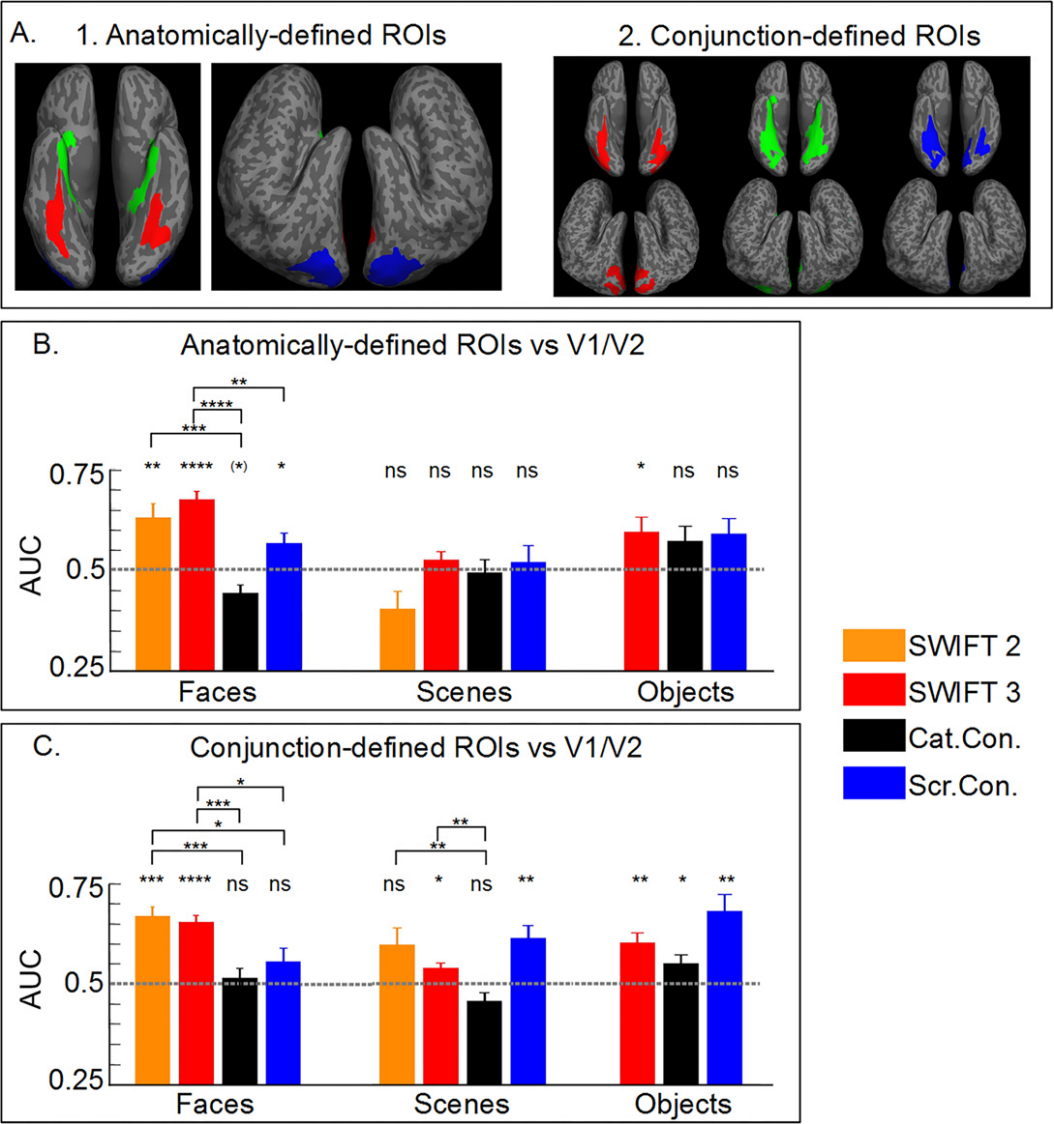
SWIFT activates the high-level areas more preferentially than the low-level areas, confirmed by criterion-free ROC analyses. (A1) Anatomically-defined and (A2) Conjunction-defined ROIs in a ventral and a lateral view. ROIs for faces, scenes and objects are represented by red, green and blue, respectively. (B, C) Area under the curve (AUC) for (B) anatomically- and (C) conjunction-defined ROIs. Relative activation between the high- and low-level areas was assessed by the ROC analyses. Hits and false alarms are defined as the proportion of significant voxels in the anatomically-defined ROIs (e.g., fusiform gyrus for faces) and in V1 and V2, respectively. Asterisks above the bars indicate either significant differences from AUC = 0.5 (one-sample t-test) or differences among different conditions (two-sample t-test, see Fig 4 legend for details).

We first defined the ROIs purely based on anatomy so that defined anatomical areas would contain highly category-selective voxels (Fig 6A1, See Materials and Methods for details). Note that the proportion of significant voxels is lower for the anatomically-defined ROIs than for the functionally-defined ROIs (e.g., Fig 4) because we intentionally included non-selective voxels for the anatomically-defined ROIs.

Fig 6B shows AUC, quantifying the relative activation of anatomically-defined ROIs and V1/V2 in a criterion-free manner, for SWIFT and the localizer (both the category and the scramble contrasts). AUCs for SWIFT were significantly above 0.5 for faces and objects, but not for scenes. This indicates that, for scenes, SWIFT activated similar proportions of voxels in parahippocampal areas and V1/V2 across different thresholds. Interestingly, only the scramble-contrast localizer's AUC was significantly greater than 0.5 for faces while, AUC for the category contrast was significantly below 0.5 for faces indicating that the category contrast resulted in more significant voxels in V1/V2 than in the fusiform gyrus over a range of thresholds. AUCs for SWIFT were significantly larger than those for the localizer with faces (see Fig 6B for details).

Our second way to neutrally define the ROIs was to select the voxels in the high-level category-selective areas activated by both SWIFT and the localizer, which we called ‘conjunction-defined ROIs’ (Fig 6A2 see Materials and Methods for details).

Considering the areas that were activated by both methods, the pattern of results (Fig 6C) was largely comparable to those presented in Fig 6B. AUCs for SWIFT were significantly larger than 0.5 for faces, scenes and objects, while those for the localizer were significantly larger than 0.5 for scenes and objects. AUCs for SWIFT were significantly larger than those for the localizer with faces and scenes (for the details see Fig 6C).

In summary, using two distinct ways to neutrally define the high-level category-selective ROIs, our criterion-free ROC analyses (Fig 6) concluded that SWIFT preferentially engaged the high-level areas over the low-level areas, more so than the localizer. This is consistent with the results in Fig 5 which showed that SWIFT increasingly activated voxels along the visual hierarchy while the localizer activated roughly the same proportion of the voxels along the visual pathway. These results clearly concluded in the positive to our second main question; SWIFT constantly activated the low-level visual areas and periodically activated the high-level visual areas

## Discussion

### SWIFT tracks category-related BOLD signals using frequency-tagging

Using SWIFT, we successfully frequency-tagged BOLD signals in a category selective way. SWIFT activations in the FFA and the PPA were highly specific for faces and scenes, respectively, discriminating well above chance among different categories (Fig 4C). Remarkably, SWIFT discriminability for faces among other categories was not different from the localizer. Importantly, our results showed that, in addition to being selective among categories, SWIFT frequency-tagged activations were largely missing in V1 and V2 (Figs 2, 5 and 6), indicating its selectivity for high-level areas.

While other methods based on phase-scrambling in the Fourier domain have been developed to study object recognition (see for example: [39]) and used in combination with fMRI to identify category-related responses [40,41], SWIFT has a critical advantage as it conserves low-level image’s properties more rigorously. While Fourier based methods scramble spatial frequency-specific information across the entire image, the wavelet-based method used in SWIFT conserves the spatial frequency content locally. We believe this is critical to maintain V1 and V2 steadily activated as any difference in spatial frequency content among retinotopic locations within the scrambled frames would be inevitably reflected as spurious frequency-tagged activations.

Previous results have shown that SWIFT activations are highly sensitive to conscious recognition, and strongly modulated by attention [10], implying that SWIFT frequency tags high-level representations. The results presented here extended the previous study, showing that the activations elicited by SWIFT are anatomically selective and specific to high-level visual areas.

While frequency-tagging has been routinely applied to retinotopic mapping in fMRI (i.e., [23,24]), to the best of our knowledge, our study is the first to frequency-tag category-related responses. Most of previous frequency-tagging studies in fMRI have employed block designs with tagging-frequencies above the temporal resolution of BOLD, thus not directly tagging BOLD signals themselves [42–47], see however [48].

Our successful BOLD category selective frequency-tagging opens up a new venue for fMRI research. In particular, a phenomenon called intermodulation between the two tagging frequencies has been successfully employed as a marker of integrative neuronal processing with EEG/MEG [49–51]. Combined with the great spatial resolution of fMRI, frequency intermodulation of frequency-tagged BOLD signals could provide further insights into how various aspects of sensory signals are integrated at finer spatial scales.

### SWIFT versus the localizer

Across stimulus categories, the activation patterns by SWIFT were more similar to the patterns obtained by the scramble- than the category-contrast from the localizer data (Fig 3B). This is expected as SWIFT implicitly contrasts activation induced by the original image with the images scrambled in the wavelet-domain. Note that SWIFT is advantageous relative to the localizer as it rigorously preserves the image’s spatial frequency content (in contrast with the box scrambling procedure which is known for introducing artifactual high-frequency components at the box borders). Nonetheless, rigorous low-level image feature preservation can also be achieved employing carefully low-level matched scrambles in a block design.

A unique and non-trivial advantage of SWIFT is that it achieves the steady baseline activation by a large set of scrambled images that uniformly sample different combinations of the identical set of locally-conserved low level-features. This can be appreciated on the time course of the relative image similarity in Fig 1C. Through one cycle of 200 frames, the amount of scrambled images that sets the baseline activation outnumbers the original image. While keeping the local low-level features constant, each of many scrambled images is maximally different to each other because each is constructed by scrambling low-level features at harmonically related temporal frequencies in the wavelet domain. Because of this property, SWIFT achieved constant activation in V1/V2. If we were to use a "particular" scrambled image from SWIFT and contrasted it with the intact image, we would have picked up much differential activation in V1/V2, similar to the results we obtained with the scramble- or category- contrast localizer.

### Differences among categories in SWIFT 2 and 3

Of the two main aims of this study, our primary interest was to test if SWIFT can periodically activate high-level areas while keeping the low-level areas constant. Accordingly, the other aim, whether SWIFT can activate the high-level area as specifically and selectively, was of less importance. We thus used a limited number of image exemplars in our experiment (n = 9), which was sufficient to address the primary question. The smaller set of instances per stimulus category makes it difficult to compare our results with those obtained with more variable sets of stimuli. Nonetheless, we believe it merits some discussion as to the difference we observed among categories in SWIFT 2 and 3 experiments, keeping in mind that some of the effects reported here might be due to our specific stimuli.

While general results with faces were remarkably similar between SWIFT 2 and 3 (Figs 3, 4 and 6), those with scenes were not. Our psychophysics results imply that the differences were unlikely to be due to perceptual difference between SWIFT 2 and 3 for scenes. One possible explanation for the difference is that the scene-related periodic activations in SWIFT 3 were reduced across the cortical areas (Fig 3A) by the introduction of the object category. As object images share low-level image features (e.g., high spatial frequency content) with scene images, those low-level features might contribute to increase the sustained ‘baseline’ activity (i.e., not frequency-tagged responses). If the baseline activity is raised, the evoked response at the tagging-frequency for scenes would be reduced in SWIFT 3 compared with SWIFT 2. This would lead to less periodic activation within PPA in SWIFT 3 than SWIFT 2 (Fig 3C). It would also lead to reduced periodic activation in V1/V2, explaining the higher normalized proportion of activated voxels in high-level areas in Fig 5D in SWIFT 3 than SWIFT 2. Consistent with this idea, previous studies showed that low-level features activate the PPA [52,53].

Relatedly, while the results with SWIFT were compelling for faces, and to a lesser extent, for scenes, those for objects were not (Figs 3–6). While this could be in part explained by our psychophysical results showing the differences in vividness between face and the other categories, here we consider two alternative explanations.

The first explanation is due to heterogeneity of our pictures in the object category (i.e., keys, a radio and a car). This could yield a poor category response as each image would activate different subpopulations within the LOC. A more homogeneous set of objects, such as tools, might have produced more compelling responses.

Another possible explanation might be that cortical modules that represent the object categories in an invariant manner may be less robust compared to those for faces. For the face category, converging evidence from clinical, intracranial recordings and neuroimaging data suggests the existence of invariant face representations in the FFA (e.g., [17,54,55]). For scenes and objects, the existence of invariant representations is less established. For example, the parahippocampal "place" area (PPA) has been shown to respond preferentially to high spatial frequencies [52] and selectively to texture information [53] that are found in scene pictures. Similarly, object-selective responses have been shown to emerge from the activation of lower-level features coded in the posterior part of the ventral pathway [56,57].

While our sub-optimal results with scenes and especially objects are consistent with the above mentioned studies, the response properties of these high-level category-selective areas are highly debated and under active investigation. Invariant neural responses in face-selective areas have been re-evaluated by studies showing strong influence of low-level properties in the face responsive areas [58–60]. On the other hand, there is evidence that support an idea that scenes and objects are represented in clusters of occipital areas, whose activity is causally related to perception of scenes and objects [61,62]. More careful studies are needed to fully understand the exact reasons why we observed the difference in response patterns among categories in SWIFT 2 and 3.

### SWIFT reveals an increase in neuronal tuning for categories along the visual hierarchy

SWIFT engaged the ventral visual pathway gradually, with the weakest responses in the early visual areas and the strongest in the category-selective areas. This confirmed our design principle of SWIFT and it is in agreement with models of object representations which predict a gradual emergence of selectivity and invariance of category representations along the ventral visual pathway [2,63–65], which has been supported by experimental data [8,56,66,67].

The most likely neural mechanism underpinning our results is gradual refinement of downstream readout in tuning. It is plausible that neurons in V3 and V4 are more preferentially activated only when a particular subset of neurons in V1 and V2 are activated, especially at the time when the original image is revealed. Given that fMRI can be more sensitive to synaptic inputs to a given area than output spikes from the area [68], our results may need to be carefully interpreted. For example, the periodic activation seen in V3 may reflect periodic output spiking from V2, which was driven by periodical refined readout of V1 neurons. This idea is consistent with a recent finding that V2 neurons are more tuned to natural image statistics than V1 [69].

These results are important for the validation and refinement of current models of visual perception. In addition, SWIFT opens new avenues to resolve longstanding questions on the functional architecture of the visual cortex.

One question concerns the role of horizontal connections in the coding of natural images. Horizontal connections, especially in V1, are implicated in contour processing [70] and they are expected to be activated periodically by the unscrambled SWIFT frame due to its coherent long-range contours, which is not what we found. We speculate that the minimal periodic engagement of V1 (Fig 2A) implies that the horizontal connections within V1 might contribute to BOLD signals in a limited way.

In the same vein, feedback activation has been shown to modulate the early visual areas via top-down attention [71–73] and, it has been suggested to be especially important for object recognition [74–77]. Again, feedback connections are expected to be activated periodically by the unscrambled SWIFT frame due to its clear object semantics.

Future fMRI studies may be able to isolate subtle horizontal and feedback activations generated by SWIFT and thus better understand the role of these connections in the coding of natural images. This can be done, for example, by utilizing directed functional connectivity measures, such as Granger causality [78] and dynamic causal modeling [79]. Intracranial layer-resolved recordings can also be used to test whether a subset of V1 neurons are periodically activated by SWIFT either via horizontal or feedback connections.

## Supporting Information

S1 Movie Example of a SWIFT stimulus showing a face and a scene. (GIF)

S1 Supporting Information Receiver operating characteristic analysis simulation. (PDF)
