# Supplementary figures and images for: Semantic Wavelet-Induced Frequency-Tagging (SWIFT) Periodically Activates Category Selective Areas While Steadily Activating Early Visual Areas
Source: PLoS One. 2015 Dec 21;10(12):e0144858. doi: 10.1371/journal.pone.0144858 (PMC4686956; doi:10.1371/journal.pone.0144858)

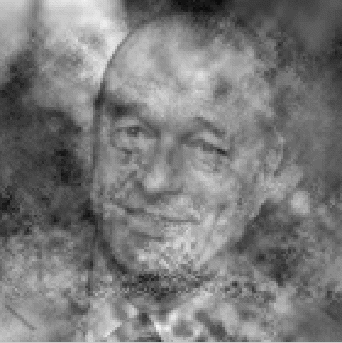

Supplement: S1 Movie — (GIF) [file pone.0144858.s001.gif]
