# Supplementary material for: Semantic Wavelet-Induced Frequency-Tagging (SWIFT) Periodically Activates Category Selective Areas While Steadily Activating Early Visual Areas
Source: PLoS One. 2015 Dec 21;10(12):e0144858. doi: 10.1371/journal.pone.0144858 (PMC4686956; doi:10.1371/journal.pone.0144858)
Supplement: S1 Supporting Information — (PDF) [file pone.0144858.s002.pdf]

Receiver operating characteristic analysis simulation

To characterize our novel ROC analysis, we performed a simulation. We constructed two ROIs, ROI1 and ROI2. For i-th iteration of simulation, we assigned a random vector emulating noisy BOLD response pattern for ROI1,

$$X(1,i)=[x(1,i), x(2,i), ... x(N,i)],$$

where N=1000 representing 1000 voxels in ROI1 and each x is distributed uniformly between 0 and 1. Similarly, we constructed a random vector for ROI2 (X(2,i)). These served as baseline responses for ROI1 and ROI2. Next, we added a ‘sensitivity’ term ranging from 0 to 1 in steps of 0.1 to ROI1 to obtain the response to a certain stimulus category,

$$Y(1, i)=X(1, i) + \text{sensitivity for ROI1},$$

Similarly, we also constructed RO2's responses. This was repeated for 15 times (i=1, 2, ...15). Then we obtained a p-value for each of 1000 voxels by performing a t-test comparing X(1,:) and Y(1,:) (degrees of freedom = 14), testing the significance of response the stimulus category at a given sensitivity in ROI1. Similarly, we obtained a p-value vector for ROI2. Using the same procedure as in Figure 4a, these p-value vectors were used to construct the ROC curves in Figure S1 at each combination of sensitivity values for ROI1 and ROI2.

The figure below shows that when ROI1’s sensitivity is higher than ROI2’s, the area under the curve (AUC) is above 0.5 (light green to red lines), whereas when ROI1's sensitivity is lower than ROI2’s, the AUC is below 0.5 (pale blue to blue lines). When both are equal (green), the AUC is 0.5. This confirms the validity of our ROC procedure.

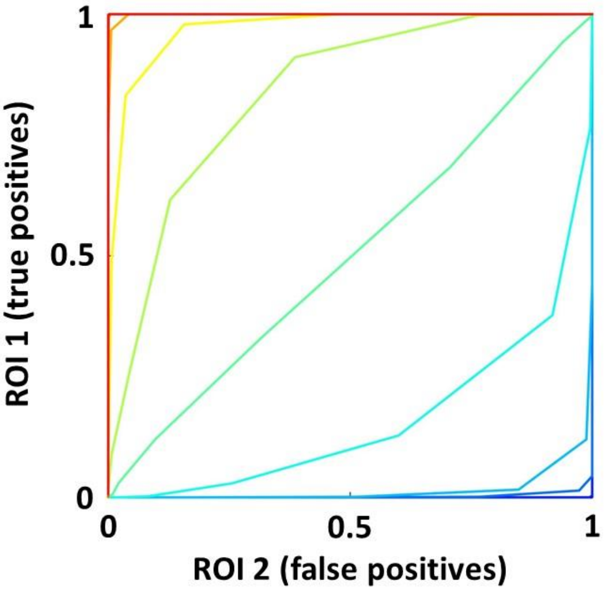

ROI 1 sensitivity	ROI 2 sensitivity	AUC
0	1	0
0.1	0.9	0.0001
0.2	0.8	0.0022
0.3	0.7	0.0279
0.4	0.6	0.1734
0.5	0.5	0.5250
0.6	0.4	0.8207
0.7	0.3	0.9732
0.8	0.2	0.9982
0.9	0.1	0.9999
1	0	1

Receiver operating characteristic analysis simulation
